# Role of graphene concentration on electrochemical and tribological properties of graphene-poly(methyl methacrylate) composite coatings

**DOI:** 10.1177/00219983231194901

**Published:** 2023-08-14

**Authors:** Amir Reza Salasel, Sukanta Bhowmick, Reza Riahi, Ahmet T Alpas

**Affiliations:** Department of Mechanical, Automotive and Materials Engineering, 8637 University of Windsor, Windsor, ON, Canada

**Keywords:** Graphene nanoplatelets, poly(methyl methacrylate), polymer-matrix composites, electrochemical properties, tribological properties, Raman mapping, degradation mechanisms

## Abstract

This study aims to investigate the influence of graphene nanoplatelet (GNP) concentration on the electrochemical and tribological properties of GNP-poly(methyl methacrylate) (PMMA) composite coatings. GNP-PMMA coatings were prepared with varying GNP concentrations (0.5, 1.0, 3.0, and 5.0 wt %) using the drop-casting method onto AA6061 aluminum alloy substrates. Results showed that the addition of 1.0 wt % GNP increased the tensile strength of PMMA but further increase reduced the tensile strength and fracture strain of the composites. Permeability studies indicated that 1.0GNP-PMMA had the lowest water vapour transition rate. All GNP-PMMA coatings showed a higher coating resistance and impedance modulus at the lowest frequency compared to neat PMMA with 1.0GNP-PMMA having the highest |Z|_0.01 Hz_ value in comparison to the composites with higher GNP concentrations. According to Raman mapping, an increase in the concentration of GNP in the composite resulted in the agglomeration of graphene, which caused the debonding of the graphene-PMMA interfaces and also resulted in a higher number of shear fronts and other defects on the fracture surface that reduced barrier properties of graphene. The specific wear rate of 1.0GNP-PMMA was lower than that of neat PMMA, indicating improved wear resistance. The coefficient of friction was lowest for 5.0GNP-PMMA, although this was due to a higher amount of material being transferred to the counterface. Accordingly, optimizing the GNP concentration enables the development of high-performance PMMA coatings with enhanced strength, improved barrier properties, and reduced wear rates, making them well-suited for applications such as corrosion protection and tribological coatings.

## Introduction

Both internal combustion engine vehicles (ICEVs) and electric vehicles (EVs) can benefit from incorporating lightweight aluminum alloys into various components In ICEVs, fuel lines play a crucial role in transporting fuel between the fuel tank, engine, and under-the-bonnet area. Typically, these fuel tubes are made from single-wall low-carbon steel tubing due to its easy formability and low raw material cost. However, lightweight Al-Mg-Si alloys, such as AA 6061, are being considered as an alternative. These components are, however, prone to corrosion and damage during vehicle operation. In EVs, battery enclosures can be made from conventional and new Al-Si-Mg based alloys. However, the exterior surface of these enclosures can be subjected to corrosion due to road salts and wear due to stone impingement. While aluminum alloys are generally corrosion-resistant, exposure to harsh environmental conditions such as the presence of chlorine (Cl^−^) ions can cause corrosion. Traditionally, chromate conversion coatings have been employed to protect aluminum from corrosion. However, the use of chemical treatments in the production of these coatings is not environmentally sustainable. Therefore, the development of new coatings that offer sufficient corrosion and wear resistance for aluminum alloys, without relying on environmentally aggressive chemicals, is of vital importance. Polymeric coatings have shown significant advantages in tribological and corrosion applications, as demonstrated by various tribological and corrosion tests.^1–3^ In recent years, there has been extensive research on polymeric composite coatings for corrosion mitigation. These coatings can incorporate nanoparticles such as silica, zirconia, and graphene, typically at volume fractions ranging from 0.5% to 5%.^4–6^ Graphene has numerous beneficial properties, including its sheet-like morphology, large surface area, and chemical inertness.^7–10^ The corrosion protection provided by graphene-polymer matrix composite coatings can arise from several factors but is usually attributed to the fact that corrosive species are prevented or hindered from penetrating the coating due to tortuous diffusion paths provided by graphene layers. In addition, the inclusion of graphene has been observed to enhance both the tensile strength and resistance to crack propagation in these composite coatings.^11^ The addition of graphene to polymer matrices has been shown to improve the friction and wear properties of polymer composites. This could be accomplished through the formation of a self-lubricating film at the contact interface, facilitated by the presence of graphene.^12–16^

The objective of this research is to develop a graphene-based composite using PMMA (Poly(methyl methacrylate)) that exhibits resistance to environmental degradation and possesses favorable mechanical and tribological properties. Additionally, the study aims to provide a rationale for the improved electrochemical characteristics observed in these composites. To achieve this, PMMA composite coatings reinforced with varying weight percentages (0.5%, 1.0%, 3.0%, and 5.0%) of graphene nanoplatelets (GNPs) were prepared using a solution mixing method. These coatings were then deposited onto aluminum 6061 surfaces through a drop-casting technique. The drop-casting method was selected for preparing the composite coatings due to its ability to provide controlled and uniform deposition, cost-effectiveness, and compatibility with the materials used.^17^ This method ensures precise control over coating thickness and promotes consistent properties throughout the samples. Several characterization techniques were employed to investigate the properties of the coatings. Tensile testing, water vapour transition rate measurements, electrochemical impedance spectroscopy, potentiodynamic polarization tests, and reciprocating wear tests were conducted to evaluate the mechanical strength, permeability, anti-corrosion properties, and tribological performance of the coatings. Raman spectroscopy was utilized to analyze the spatial distribution of graphene within the coatings, while scanning electron microscopy (SEM) allowed for the observation of surface defects on fractured samples. When the mechanical, electrochemical, and tribological tests were considered together, a clear and compelling finding emerged: the addition of graphene in an optimal amount yields the highest benefits across all investigated properties.

## Materials and experimental procedure

### Preparation of composite coatings

To apply the composite coatings onto the aluminum substrate, the GNP-PMMA solution was drawn into a syringe and subsequently injected onto pre-cleaned AA 6061 grade aluminum substrates. The injection was performed in the form of circular samples with a diameter of 16 mm, ensuring that the coated area was evenly distributed. Prior to coating, the aluminum substrate was cleaned using a solution of 10 wt % sodium hydroxide to remove any impurities and ensure proper adhesion of the coating. The graphene powders were obtained from Graphene Laboratories Inc., located in Reading, MA, USA. Polymethyl methacrylate (PMMA) powder and Dimethylformamide (DMF) solvent with a boiling point of 153°C were purchased from Alfa Aesar and Sigma-Aldrich, respectively. Sodium Hydroxide was purchased from ACP Chemicals Inc.

To prepare the composite coatings, different weight percentages of GNP (0.5%, 1.0%, 3.0%, and 5.0%) were incorporated into PMMA. For example, for the 1.0 wt % GNP composite film, 0.032 g of GNP was dispersed in 5 mL DMF in an ultrasonic bath for 6 h. Meanwhile, 3.168 g of PMMA was dissolved in 25 mL DMF at 35°C. The GNP/DMF suspension was then mixed with the PMMA/DMF solution and stirred for 12 h under an inert atmosphere in a glove box. This resulting coating was designated as 1.0GNP-PMMA, and the other composite coatings were prepared in a similar manner (0.5GNP-, 3.0GNP-, 5.0GNP-PMMA) along with neat PMMA. The coatings were applied onto pre-cleaned AA 6061 grade aluminum substrates using a drop-casting method. The sheets were 0.8 mm in thickness. 6061 aluminum alloy typically consists of (as weight percentages) 0.6%–1.2% magnesium (Mg), 0.4%–0.8% silicon (Si), up to 0.7% iron (Fe), 0.15%–0.4% copper (Cu), 0.04%–0.35% chromium (Cr), up to 0.25% zinc (Zn), and up to 0.15% titanium (Ti) and the balance aluminum.

A syringe was used to inject the GNP-PMMA solution onto the substrates in the form of circular samples with a diameter of 16 mm, ensuring even distribution of the coated area. Prior to coating, the aluminum substrate was cleaned with a 10 wt% sodium hydroxide solution to remove impurities and promote adhesion. The coated samples were then cured in a furnace at 60°C for 12 h to evaporate any residual solvent. A schematic flowchart of the procedures used for the preparation of the composites is shown in Appendix 1, which provides a visual representation of the step-by-step process followed in the preparation of the composite films.

The tensile test samples were prepared by depositing the composite films onto a glass sheet using a film applicator, creating free-standing films. These films were then cured at 60°C for 12 h. To facilitate the removal of the films, they were isothermally heated in a water bath at 35°C for 3 h., making it easier to peel them from the glass surfaces. The same method was employed to prepare samples for water vapor permeability tests.

### Mechanical properties of graphene-PMMA composites

To evaluate the mechanical properties of the composites, tensile tests were carried out using a desktop testing machine (Mark-10 ESM303) with a maximum capacity of 1.5 kN at a crosshead speed of 0.5 mm/min. The samples used for the tensile tests were miniature dumbbell-shaped specimens having an overall length of 29 mm, a width of grip section of 5 mm, a gauge length of 7 mm, and an outer radius of 25 mm. These samples were precisely cut using a specially manufactured steel die with knife edges, ensuring smooth edges with a thickness of 30 ±5 μm. The engineering stress-strain curves were obtained by dividing the load applied by the cross-sectional area of each specimen to calculate the engineering stress, and the engineering strain was determined from the crosshead displacement. Three tensile tests were conducted on each sample, and the typical stress-strain curves are given in the results section. The average tensile strength and strain at fracture values from the three tests were then plotted based on the GNP content of the composites.

The samples used for the tensile tests were miniature dumbbell-shaped specimens with dimensions of 29 mm length, 5 mm grip section width, 7 mm gauge length, and 25 mm outer radius. Although these samples differed from the standard ASTM D638 dimensions, they were considered “miniature” within this study’s context. This alteration allowed for the preparation of a greater number of test samples from limited composite film quantities with specific GNP contents. The samples were carefully prepared using a steel die with knife edges to ensure smooth edges and a thickness of 30 ± 5 μm. The engineering stress-strain curves were obtained by dividing the applied load by the cross-sectional area of each specimen, and the engineering strain was calculated from the crosshead displacement. Three tensile tests were performed on each sample, and the results were used to generate typical stress-strain curves. The average values of tensile strength and strain at fracture, obtained from the three tests, were then plotted as a function of the GNP content in the composites.

### Water vapour permeability

In the water vapour permeability test, the Elcometer 5100/1 Payne permeability cup with a test area of 10 cm^2^ was used. The test was conducted using distilled water as the testing medium. The test setup included distilled water, a cup, a rubber gasket, a sealing ring on the film, and the clamps. At the start of the test, the entire arrangement was weighed, and subsequent weight changes were recorded throughout the experiment.^18^ Data were collected at seven time intervals, each lasting 1 h. The test was conducted under controlled conditions of a temperature of 35°C and a relative humidity of 30%.

WVTR was obtained by following ASTM Standard E96/E96M-16^19^ and using the following equation to calculate WVTR in (g/(m^2^.d)):

(1) $$WVTR=240\times \frac{∆m}{A}$$

Where ∆m is the rate at which mass changes in mg/h and A is the area of the test piece in square centimetres.

Another indicator of a film's water vapour barrier property is its water vapour permeation, (WVP) measured in units of g⋅µm/m^2^⋅day⋅kPa. WVP was determined using the following equation:

(2a) $$WVP=\frac{WVTR.t}{DP}$$

Where *t* is the thickness of the film and DP is the difference in vapour pressure between the two sides of the film and expressed as follows:

(2b) DP=S. (R1−R2)

Where *S* is the saturation vapour pressure at test temperature, *R1* is the relative humidity at the source expressed as a fraction, and *R2* is the relative humidity at the vapour sink expressed as a fraction.

### Electrochemical measurements

The electrochemical measurements were performed using a three-electrode system in a 5% wt. NaCl solution. The temperature was controlled by using a water bath. The counter electrode was made of graphite rods, offering a larger surface area compared to the working electrode. A saturated calomel electrode (SCE) served as the reference electrode. The electrochemical impedance spectroscopy (EIS) tests were conducted using a PARSTAT 3000A instrument. The specimens were immersed in the electrolyte for 12 h prior to testing. The disturbance amplitude was 10 mV at open circuit potential, and the frequency range for all tests was 100 MHz-0.01 Hz. Bode and Nyquist plots were generated for each coating.

Potentiodynamic polarization (PDP) analysis using a DC technique was performed utilizing the Solartron system. Prior to conducting the electrochemical tests, an initial two-hour measurement of open circuit potential (OCP) was carried out to ensure a stable potential. The potentiodynamic scanning (PDS) measurements involved scanning from a cathodic potential of −300 mV to an anodic potential of 300 mV relative to EOCP, with a scanning rate of 1 mV/s. Tafel plots were extracted from these scans to scrutinize the electrochemical behaviour. The corrosion current density (I_corr_) and corrosion potential (E_corr_) were ascertained, with I_corr_ offering insight into the corrosion rate exhibited by the working electrode.^20^ Both uncoated and coated samples were used as working electrodes in the measurements.

### Friction and wear tests

The tribological performance of GNP-PMMA composite coatings was studied using a reciprocating tribometer (CSM Instruments). The tests were conducted with a constant load of 1N and a sliding velocity of 2 Hz (equivalent to 0.04 m/s) for a sliding distance of 150 m. M2 grade steel pins with rounded ends and a diameter of 4 mm were used as the counterface in the tests. The load applied during the sliding tests was intentionally kept at a mild level to induce controlled wear and maintain a steady contact pressure. This approach ensured that the applied load does not cause abrupt removal of the coating, allowing for a gradual evaluation of the coating's wear resistance and its ability to withstand sliding conditions over an extended duration. The selection of a sliding velocity of 2 Hz (0.04 m/s) was a deliberate choice to replicate the typical sliding or rubbing motion between the PMMA-based composite coating and the aluminum substrate This moderate sliding velocity emulated the relative motion experienced by PMMA coatings on aluminum surfaces and generated typical coefficient of friction for aluminum in dry wear.

Each composite was tested three times, and wear measurements were taken at the end of the 150 m distance. The wear tracks of the coated samples were analyzed using a Keyence optical microscope to obtain 3D surface profiles. The specific wear rate was calculated by measuring the volumetric wear losses using 2D surface profilometry traces at multiple locations along the wear track. The estimated volume loss for each sample was then divided by the sliding distance and the normal load to obtain the specific wear rate.

### Characterization of the composites

The cryogenically fractured (at around −80°C) surfaces of the coatings were examined using a FEI Quanta 200 FEG scanning electron microscope (SEM) operated at an accelerating voltage of 5 kV. The SEM analysis aimed to investigate the morphology and structure of the coatings. These observations were conducted to identify any defects in the polymer matrix and at the interfaces between the GNP and the matrix, with a focus on the influence of GNP percentage in the coating. Additionally, SEM was employed to examine the wear tracks and the material transferred to the counterface during the sliding wear tests.

The structure of the GNPs was analyzed using a WiTec Raman spectrometer equipped with a 3 mW solid-state laser operating at a 532.0 nm excitation line. Raman maps were generated to assess the dispersion of GNPs in the PMMA matrices by measuring the integrated intensity of the G peak exhibited by the GNPs.

## Results

### Mechanical properties of the GNP-PMMA composite coatings: Stress-strain curves

**Figure 1.**
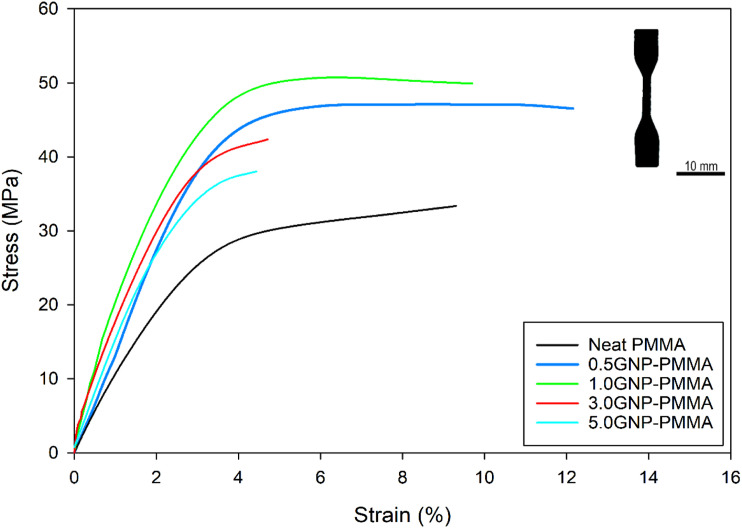
Engineering stress-strain curves for neat PMMA and GNP-PMMA composites. The inset shows the photograph of an actual tensile sample.

The inclusion of small quantities of GNP (less than 3%) has led to an enhancement in both the tensile strength and fracture strain of the PMMA composite films. Conversely, when the GNP percentage surpassed 3%, the observed trend was reversed, resulting in a reduction in both tensile strength and fracture strain. This trend is clearly depicted in the stress-strain curves presented in Figure 1 where it becomes evident that the PMMA composite films containing 0.5 and 1% GNP exhibited the highest tensile strength and fracture strain compared to the pure PMMA film, while the film incorporating 3 and 5% GNP exhibited lower values compared to the other composites tested.

**Figure 2.**
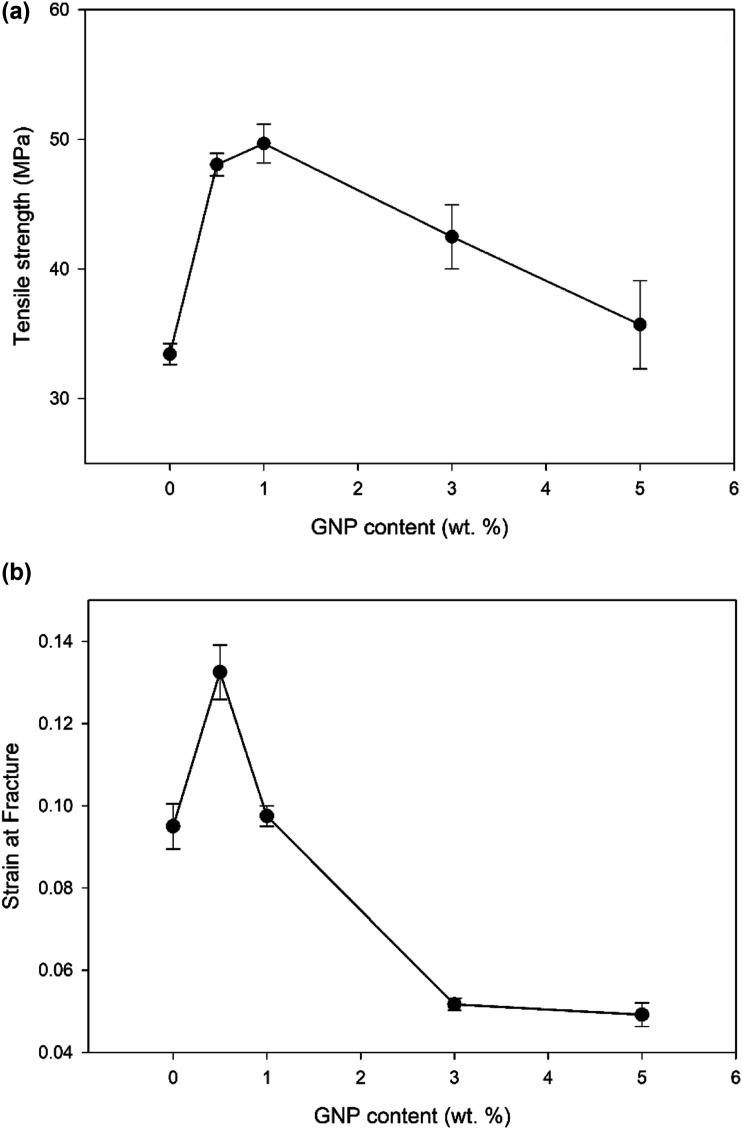
(a) Tensile strength as a function of GNP concentration (b) Strain at fracture as a function of GNP concentration.

The incorporation of 1 wt % GNP into the PMMA matrix resulted in a mean tensile strength of 49 ± 1.49 MPa, which was approximately 50% higher than the mean tensile strength of the neat PMMA sample, measured at 33 ± 0.80 MPa. However, the mechanical properties of the composite films with higher GNP concentrations (3 wt % and 5 wt %) showed a weakening trend; According to Figure 2(a), the tensile strength of the 3.0GNP-PMMA composite decreased to 43 ± 2.46 MPa, while the 5.0GNP-PMMA composite exhibited a further reduction to 36 ± 3.39 MPa compared to the 1.0GNP-PMMA composite.

The fracture strain of the neat PMMA samples was found to be 0.095 ± 5.492 × 10^−3^. Upon the addition of 0.5 wt % GNP, the fracture strain of the composite films increased to 0.1325 ± 6.614 × 10^−3^. However, as the GNP concentration was further increased to 1.0 wt %, 3.0 wt %, and 5.0 wt %, the fracture strain decreased to 0.0975 ± 2.500 × 10^−3^, 0.0516 ± 1.443 × 10^−3^, and 0.0491 ± 2.886 × 10^−3^, respectively. These results, as shown in Figure 2(b), indicated that the addition of a low GNP concentration (0.5 wt %) contributed to an increase in fracture strain, but higher GNP concentrations resulted in a decrease in fracture strain below that of the neat PMMA samples. The reduction in fracture strain was attributed to the agglomeration of the graphene plates. Raman spectroscopy mapping showed that at 3% GNP concentration, graphene flakes agglomerated, indicating a high degree of clustering. When larger concentrations of GNP were added to the PMMA matrix, the GNPs began to cluster or agglomerate, creating areas of stress concentration and reducing the ability of the material to deform before breaking. Interfacial cracks were observed as well further contributing to the decrease in fracture strain. The load transfer between PMMA and graphene plates was most effective when the graphene was uniformly dispersed in the matrix. These phenomena will be explained in more detail later in the manuscript.

### Water vapour permeability of the GNP-PMMA composite coatings

**Figure 3.**
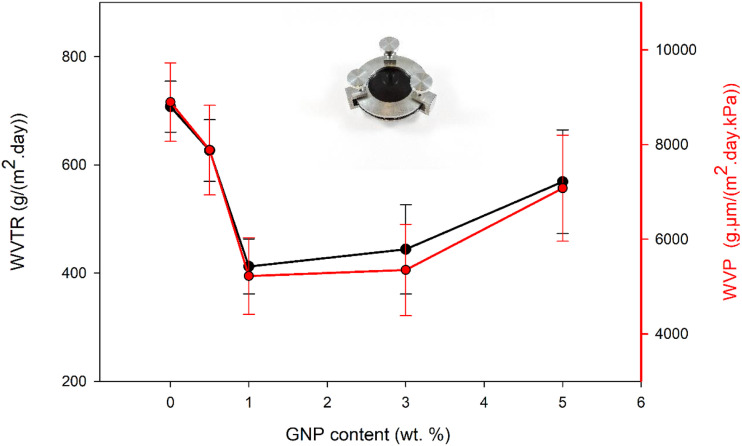
WVTR and WVP of neat PMMA and GNP-PMMA composite films. The inset shows the photograph of a permeability cup made of anodised aluminum and used to determine the water vapour permeability.

Figure 3 shows the effect of GNP concentration on the gas barrier properties of the PMMA matrix. Specifically, the incorporation of 0.5 wt% and 1 wt% GNP into the PMMA matrix led to a decrease in both the water vapor transmission rate (WVTR) and water vapor permeability (WVP), indicating enhanced barrier properties. Notably, the lowest WVTR and WVP values were observed for the 1.0GNP-PMMA composite.

The incorporation of 1 wt% GNP in the PMMA matrix led to a reduction in WVTR from 707.66*g*/(m2.d) for neat PMMA to 412.19*g*/(m2.d). This significant decrease in WVTR demonstrates the effectiveness of introducing GNPs at this particular concentration to enhance the gas barrier performance of the composite. The diminished WVTR signifies a decrease in the permeability of water vapour through the composite film, indicating an improved capacity to resist the infiltration of moisture.

However, the incorporation of 3 wt % and 5 wt % GNP was shown to increase the diffusion of water molecules leading to an increase in the values of WVTR and WVP. Specifically, the WVTR of 3.0GNP-PMMA and 5.0GNP-PMMA increased to 444*g*/(m^2^.d) and 569*g*/(m^2^.d). The results so far have shown that the addition of small amounts of GNP (up to 1 wt %) to PMMA improved both the mechanical and barrier properties of the composite films, while the addition of larger amounts of GNP (3 wt % and 5 wt %) can compromise the mechanical properties and diminish the composite’s ability to impede the transmission of water molecules.

### Corrosion testing of the GNP-PMMA composite coatings

**Figure 4.**
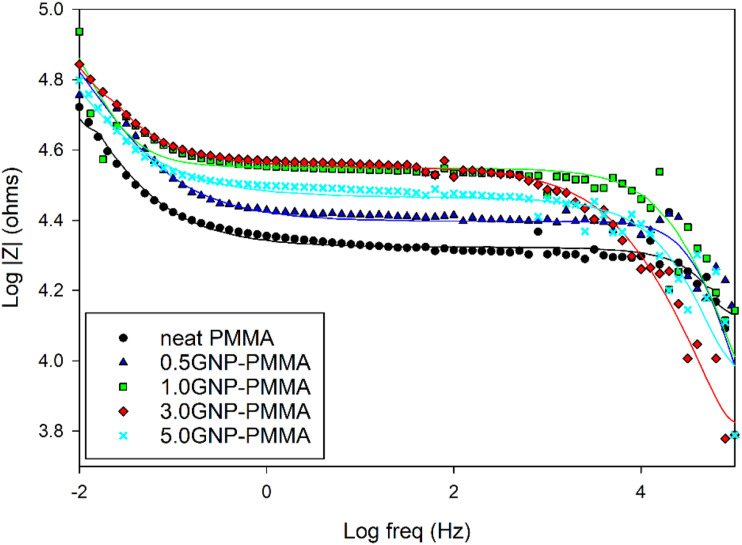
Bode plot of neat PMMA and GNP-PMMA composite coatings after immersion in 5 wt % NaCl solution for 12 h.

The anti-corrosion performance of the GNP-PMMA composite coatings was evaluated using the EIS (Electrochemical Impedance Spectroscopy) technique. Bode and Nyquist plots were generated for coatings with various GNP concentrations to assess their corrosion resistance. In Figure 4, the Bode plot is presented, showing the variation of the logarithm of impedance modulus (|Z|) with the logarithm of frequency for both the neat PMMA and GNP-PMMA samples. The measurements were conducted in a 5 wt % NaCl solution for a duration of 12 h. The corrosion resistance is determined by the impedance modulus, Z, at the lowest frequency (|Z|f= 0.01 Hz).^21^ At this low frequency, the corrosion process is considered to occur at the interface between the aluminum substrate and the coating.^22–24^ A higher value of log Z at the lowest frequency indicates a high impedance, meaning that the system is more resistant to the flow of electric current. This could be the case when GNP is present in the coating and acting as a barrier to corrosive species. If agglomeration of GNP reduces the barrier properties and increases conductivity, this would likely lead to a decrease in the impedance and a shift of the Bode plot towards lower values of log Z. The specific changes on the Bode plot would depend on the properties of the coating and the interactions between the GNP and the electrolyte solution. As shown in Figure 4, GNP-PMMA coatings exhibited a greater Z-modulus at 0.01 Hz in comparison with that of the neat PMMA coatings. The values of |Z|_0.01 Hz_ for all tested coatings are presented in Table 1, where it is evident that the composite containing 1 wt % GNP had the highest |Z|_0.01 Hz_ value compared to the other composites. However, the value of (|Z|_0.01 Hz_) decreased as the concentration of graphene in the PMMA matrix was increased.

**Table 1.** EIS parameters for neat PMMA and GNP-PMMA composite coatings, including impedance modulus at the lowest frequency, coating and charge transfer resistance, constant Y_0_ and exponent n for coating and double layer constant phase element.

Sample	|Z|_0.01 Hz_ ( Ω )	R_coat_ ( Ω )	R_CT_ ( Ω )	CPE_coat_ (Y_0_) ( $\Omega^{-1}{cm}^{-2}s^{n}$ )	*n*_1_	CPE_dl_ (Y_0_) ( $\Omega^{-1}{cm}^{-2}s^{n}$ )	*n*_2_
Neat PMMA	53028.70	21401	1.16 × 10^5^	2.60 × 10^−10^	0.9272	1.86 × 10^−4^	0.7448
0.5GNP-PMMA	58493.65	24947	1.21 × 10^5^	1.07 × 10^−10^	0.9960	1.64 × 10^−4^	0.6431
1.0GNP-PMMA	85113.82	37105	1.84 × 10^5^	1.04 × 10^−9^	0.7340	1.82 × 10^−4^	0.8052
3.0GNP-PMMA	69753.17	35214	1.68 × 10^5^	4.77 × 10^−9^	0.7253	1.51 × 10^−4^	0.7459
5.0GNP-PMMA	62825.96	29164	1.58 × 10^5^	2.23 × 10^−9^	0.6615	1.61 × 10^−4^	0.7822

**Figure 5.**
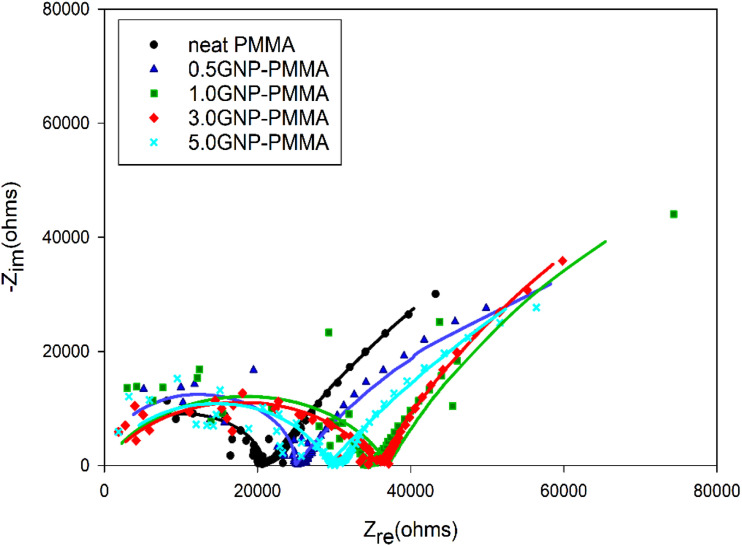
Nyquist plot of neat PMMA and GNP-PMMA composite coatings obtained after immersion in 5 wt % NaCl solution for 12 h.

The Nyquist plot for the *GNP-PMMA* samples is shown in Figure 5. The plot displays the impedance response of the samples at various frequencies. In the low-frequency region of the plot, the observed behavior corresponds to corrosion reactions taking place at the interface between the metal substrate and the coating. On the other hand, the high-frequency region reflects the impedance of the coating itself.^25^ The occurrence of a semicircle in the low to intermediate frequency range corresponds to the charge transfer resistance. The size and characteristics of this semicircle provide valuable insights into the dispersion of GNPs and their effect on the corrosion protection properties of the GNP-PMMA composite coatings.

When the GNPs are well dispersed and act as a barrier, the impedance arc in the Nyquist plot is expected to be larger. This is attributed to the well-dispersed GNPs forming a uniform and continuous barrier layer, effectively reducing overall corrosion and increasing the impedance of the system. The larger semicircle indicates a higher charge transfer resistance, signifying improved corrosion protection. Conversely, when the GNPs are agglomerated or poorly dispersed, they can create pathways for corrosive species to reach the metal substrate. This results in an increase in the corrosion rate and a smaller semicircle in the Nyquist plot. The reduced size of the semicircle indicates a lower charge transfer resistance, indicating inadequate dispersion and compromised corrosion protection.

**Figure 6.**
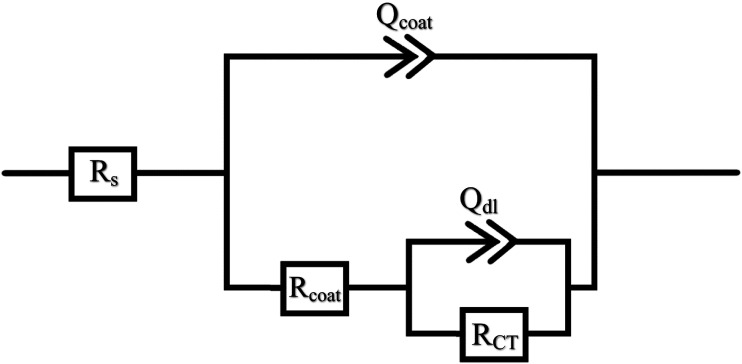
Equivalent circuit used for fitting the data of GNP-PMMA composites.

The experimental EIS data was fitted using EIS Spectrum Analyser software, and a representative electrochemical equivalent circuit was constructed, as shown in Figure 6. This equivalent circuit comprises several elements aimed at representing the electrochemical behaviour of the system.^26^ The elements in the circuit include the solution resistance (R_s_), which accounts for the resistance of the electrolyte solution in which the samples are immersed. The coating resistance (R_coat_) represents the resistance offered by the protective coating on the metal substrate. The coating constant phase element (Q_coat_) is a component that captures the non-ideal capacitive behaviour of the coating, indicating deviations from an ideal capacitor. The charge transfer resistance (R_CT_) is a crucial element that reflects the resistance to charge transfer at the metal-coating interface. This resistance is associated with the electrochemical reactions occurring during corrosion processes. The double layer constant phase element (Q_dl_) represents the behaviour of the electrical double layer formed at the metal-coating interface. The constant phase element (CPE) is employed to account for the non-ideal capacitive behaviour of certain elements in the circuit. The exponent ‘n' of the CPE can vary between 0 and 1, where *n* = 1 signifies ideal capacitor behaviour. The value of ‘n' provides insights into the deviation from ideal dielectric behaviour, with *n* = 0 indicating ideal resistor behaviour.^26–28^ A constant phase element has the following impedance:

(3) $$Z_{CPE}=\frac{1}{Y_{0}{\left(j\omega \right)}^{n}}$$

Where Y_0_ is the constant of CPE, and ω is the frequency in rad/s.^28^

Figure 5 shows that the damage due to degradation potentially in the form of the formation of voids and cracks at the GNP polymer interfaces (as will be discussed later) can be represented by R_coat_,^29,30^ while R_CT_ assesses the electrical resistance to the transfer of electrons at the interface between the aluminum substrate and the coating.^31^ The results in Table 1 indicate that all composite coatings have increased R_coat_ and R_CT_,^28,32^ compared to the neat PMMA coating, with the 1.0 wt% GNP composite displaying the highest values. These increases in R_coat_ and R_CT_ are due to the barrier properties of graphene plates, which decrease the diffusion of electrolytes to the metal/coating interface.^33–35^ However, the incorporation of 3.0 and 5.0 wt% GNP decreased the values of R_coat_ and R_CT_, which was attributed to the agglomeration of the graphene plates reducing their efficiency as corrosion barriers. These findings were further discussed in the section.

**Figure 7.**
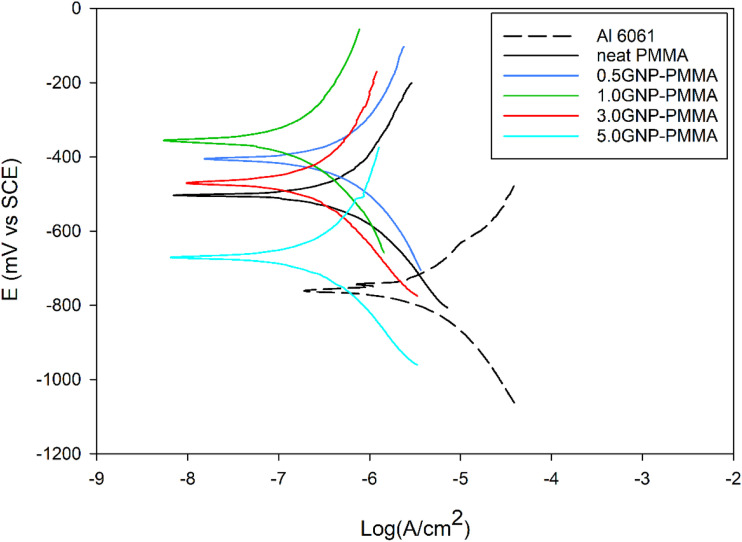
Tafel polarization curves uncoated AA6061, neat PMMA, 0.5GNP-PMMA, 1.0GNP-PMMA, 3.0GNP-PMMA, and 5.0GNP-PMMA composite coatings.

Figure 7 shows the Tafel polarization curves of uncoated 6061 aluminum, neat PMMA, 0.5GNP-PMMA, 1.0GNP-PMMA, 3.0GNP-PMMA, and 5.0GNP-PMMA composite coatings tested in NaCl solution (5.0 wt %). The potential is plotted as a function of the logarithm of the current density. By extrapolating the cathodic and anodic polarization curves and finding their intersection, the corrosion potential and corrosion current can be obtained. The data from the polarization curves are listed in Table 2, which includes the current density (I_corr_), the corrosion potential (E_corr_), the inhibition efficiency ( η ), and the calculated corrosion rate (CR).

**Table 2.** Measurements of electrochemical parameters based on potentiodynamic polarization test, including corrosion potential and current density, corrosion rate with a unit of Mils per year, and inhibition efficiency.

Sample	$E_{corr}\;\left(mV\right)$	$I_{Corr}\left(\frac{A}{{cm}^{2}}\right)$	CR (mpy)	IE (%)
Aluminum 6061	−771.1 ± 5.95	$3.08\times 10^{-6}\pm \;2.55\times 10^{-7}$	1.33	—
Neat PMMA	−504.36 ± 5.51	$4.75\times 10^{-7}\;\pm \;2.03\times 10^{-8}$	0.21	84.21
0.5GNP-PMMA	−406.48 ± 34.54	$3.24\times 10^{-7}\pm \;2.14\times 10^{-8}$	0.14	89.47
1.0GNP-PMMA	−367.73 ± 17.14	$1.13\times 10^{-7}\;\pm \;1.75\times 10^{-8}$	0.05	96.24
3.0GNP-PMMA	−451.26 ± 15.82	$2.05\times 10^{-7}\;\pm \;2.41\times 10^{-8}$	0.09	93.23
5.0GNP-PMMA	−666.93 ± 19.70	$2.74\times 10^{-7}\;\pm \;3.04\times 10^{-8}$	0.12	90.98

I_corr_ values were used to calculate corrosion rates and inhibition efficiency η using the following equations.

(4a) $$CR\;\left(mpy\right)=\frac{0.129\;.\;I_{Corr}\;.\;EW}{\rho }$$

(4b) $$\frac{I_{Corr}-I_{Corr\left(i\right)}}{I_{Corr}}\times 100$$

where the corrosion current density of the uncoated substrate and the coating is designated as I_corr_ and I_corr(i)_. EW and ρ are equivalent weight and density of sample, respectively.

It can be seen that PMMA-coated substrates showed considerably lower I_corr_ than uncoated 6061 substrates. With the introduction of GNP into the PMMA matrix, I_corr_ was further decreased and η increased. Polymer coatings often have pores and pinholes as defects. It was suggested that corrosion-inducing ions can use the internal pores and defects inside the coatings as diffusion paths causing acceleration of corrosion rate on the metal substrates.^37,38^ Large graphene flakes in the matrix may act as physical barriers to diffusion. These mechanisms are extensively discussed qualitatively in the literature.^39–43^ The 1.0GNP-PMMA showed the lowest I_corr_. However, the value of I_corr_ and η changed in the opposite direction after more graphene was added. The E_corr_ of 1.0GNP-PMMA also showed a shift towards more positive values as compared to neat PMMA and 0.5GNP-PMMA. However, GNP contents of 3.0 wt% and 5.0 wt% resulted in higher corrosion current densities compared to PMMA with 1.0 wt% GNP (but still lower than neat PMMA and 0.5GNP-PMMA). Hence, 1.0GNP-PMMA composite coatings offered the highest corrosion resistance compared to other coatings.

In summary, the addition of GNP to the PMMA matrix significantly improved the corrosion resistance of the coatings compared to neat PMMA. The 1.0 wt% GNP composite exhibited the highest corrosion resistance among all the coatings tested, with the lowest corrosion current density and the highest inhibition efficiency. The incorporation of higher GNP contents (3.0 and 5.0 wt%) decreased the corrosion resistance, attributed to the agglomeration of graphene plates reducing their efficiency as corrosion barriers. The Tafel polarization curves showed that GNP acted as physical barriers to diffusion and reduced the corrosion-inducing ions' ability to use the internal pores and defects inside the coatings as diffusion paths. The defects that can occur during the manufacturing process of coatings can significantly affect their performance. To investigate these defects, several techniques can be used, including Raman mapping and cryogenic fracture surface investigations. Raman mapping is a technique that uses laser light to probe the molecular structure of a sample. It can provide information on the distribution of various molecular species in a material. In the case of coatings, Raman mapping can be used to identify any inhomogeneities in the coating layer that could lead to defects. For example, Raman mapping can detect regions with a high concentration of graphene flakes that could potentially reduce the effectiveness of the coating as a barrier against corrosion. Cryogenic fracture surface investigations involve cooling a sample to very low temperatures and then fracturing it. This technique can provide information on the fracture surface and can be used to identify any defects, such as cracks or voids, that may have formed during the manufacturing process or testing. By analyzing the fracture surface, it is possible to determine the type of defect that occurred and its likely cause. The findings from these analyses can potentially be applied to enhance the manufacturing process and develop more effective coatings with fewer defects. Further discussion on these findings will be provided in the upcoming section. A detailed description of these findings will be presented in the ensuing “Discussion” section. Meanwhile, it is pertinent to investigate the tribological properties of the coatings.

### Tribological properties of the GNP-PMMA composite coatings

The effect of GNP on the tribological properties of the GNP-PMMA coatings was investigated by determining the values of the coefficient of friction (COF) and wear rates of composites with various graphene contents (0.5, 1, 3, and 5 wt %).

**Figure 8.**
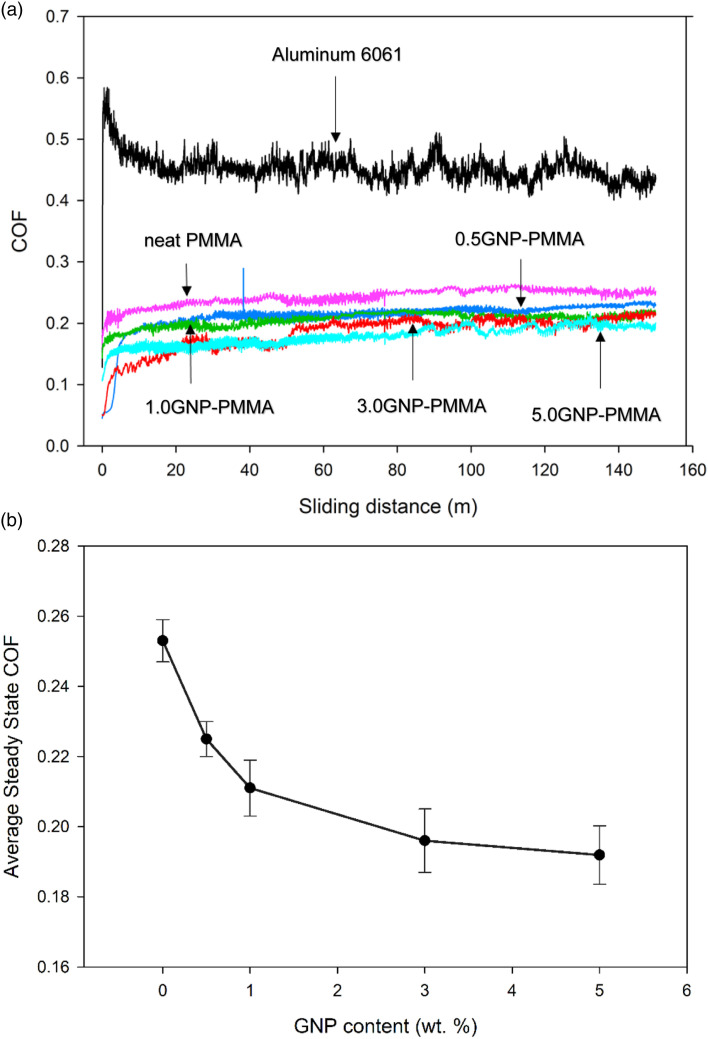
(a) COF of uncoated AA6061, neat PMMA, and GNP-PMMA composite coatings as a function of sliding distance (b) Average steady state coefficient of friction of neat PMMA, and GNP-PMMA composite coatings as a function of GNP content.

Figure 8(a) shows the changes in COF values of 6061 aluminum, neat PMMA, and polymer coatings with different GNP concentrations over the sliding distance. The COF of composite coatings increased and reached a peak during the running-in period, then stabilized at longer sliding distances. The average steady-state COF for uncoated aluminum was 0.447 ± 0.016. The average steady-state COF of neat PMMA was 0.253 ± 0.006, while the average steady-state COF of GNP-PMMA with 0.5 wt %, 1.0 wt %, 3.0 wt %, and 5.0 wt % of GNP were 0.225 ± 0.005, 0.212 ± 0.008, 0.196 ± 0.009, and 0.191 ± 0.009, respectively.

**Figure 9.**
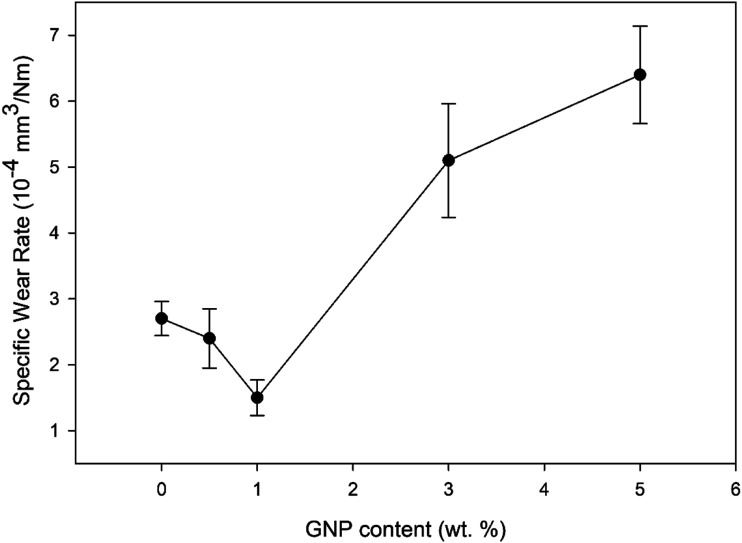
Specific wear rate for the neat PMMA and GNP-PMMA composites as a function of GNP content.

Figure 8(b) indicates that the composite coating containing 5.0 wt % graphene had the lowest COF compared to neat PMMA and the rest of the composite coatings. Figure 9 shows the specific wear rates of neat PMMA and composite coatings as a function of GNP concentrations.

**Figure 10.**
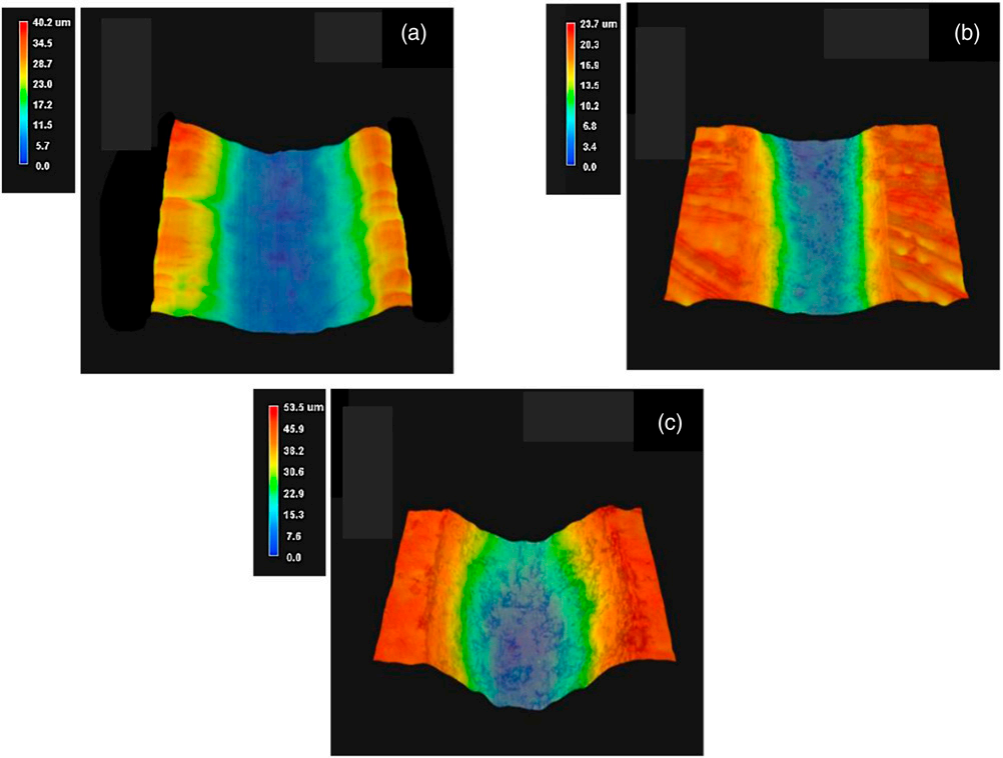
3D surface profiles of the wear tracks of (a) neat PMMA, (b) 1.0GNP-PMMA and (c) 3.0GNP-PMMA.

The neat PMMA had a specific wear rate of 2.71 ×10^−4^ mm^3^/(Nm). Incorporation of 0.5 and 1.0 wt % GNP to the PMMA matrix reduced the specific wear rates d to 2.43 ×10^−4^ and 1.58 ×10^−4^ mm^3^/(Nm), respectively. However, the specific wear rates increased significantly for the composites with higher GNP concentrations. The specific wear rates increased to 5.09 ×10^−4^ and 6.44 ×10^−4^ mm^3^/(Nm) for 3.0GNP-PMMA and 5.0GNP-PMMA, respectively. In Figure 10, 3D surface profilometer images of the wear tracks of neat PMMA, 1.0GNP-PMMA and 3.0GNP-PMMA are shown.

**Figure 11.**
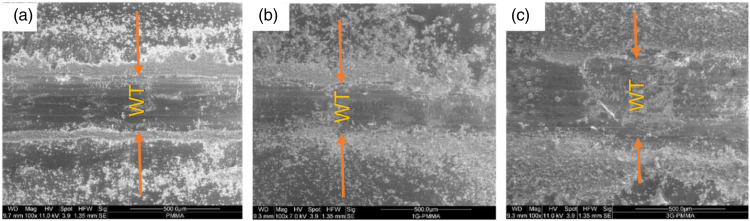
Wear tracks (WT) of (a) neat PMMA, (b) 1.0GNP-PMMA and (c) 3.0GNP-PMMA coatings.

Figure 11 shows the wear tracks of neat PMMA, 1.0GNP-PMMA, and 3.0GNP-PMMA coatings. It can be observed that the addition of 3 wt % GNP to the PMMA resulted in a considerably larger wear track width compared to neat PMMA and 1.0GNP-PMMA. This indicates that the low COF values at higher GNP concentrations were related to the higher amount of wear. The material detached from the wear tracks was likely transferred to the surface of the counterface, which reduced the friction. This phenomenon will be further investigated in *Discussion*.

## Discussion

**Figure 12.**
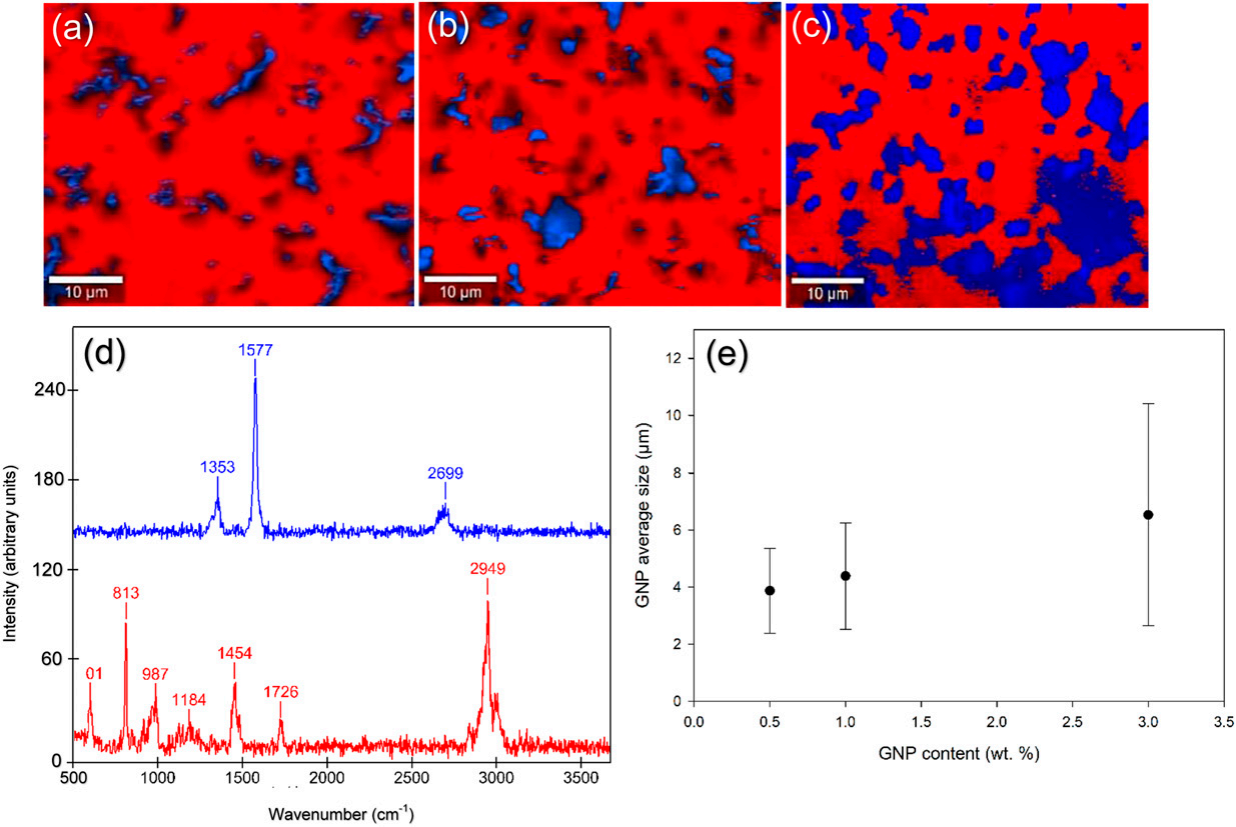
Raman maps showing the distribution of GNP (blue) in the PMMA matrix(red) obtained by (a) 0.5GNP-PMMA, (b) 1.0GNP-PMMA, and (c) 3.0GNP-PMMA (d) Raman spectra of incorporated GNPs (blue) and PMMA matrix (red), (e) relationship between the average size and the content (wt. %) of GNP agglomerates.

In this section, the focus will be on discussing the consequences of increasing the amount of GNP in the composite on its electrochemical and tribological properties. The emphasis is on investigating the variation in GNP distribution within the matrix as the GNP percentage increases, and assess its influence on the overall properties of the composite. By examining the changes in distribution, we aim to understand the impact of these variations on the composite's performance characteristics. To analyze the distribution of graphene within the composite, we employed Raman spectroscopy mapping was employed. This technique allowed us to gather spectral data to be gathered from various locations on the surfaces of GNP-PMMA composites with different GNP percentages (0.5%, 1%, and 3%). The results of this mapping are presented in Figure 12(a)–(c), providing visual representations of the distribution patterns.

The distribution of specific vibrational modes associated with the lattice structure of graphene nanoplatelets (GNP) was mapped by collecting Raman spectra at multiple locations on the surface of composites. The Raman spectra of GNP (Figure 12(d)) collected using a laser with a wavelength of 532 nm exhibited the typical peaks corresponding to the D band at 1345 cm^−1^, the G band in-plane vibration of sp^2^ hybridized carbon atoms at 1576 cm^−1^, and the 2D band at 2700 cm^−1^ due to scattering of two phonons as a result of double resonance of the G band. The 2D band is sensitive to the number of layers in the graphene, and a value of I(2D)⁄I(G)<1 indicates a multilayered structure of GNP.^44,45^ An area of approximately 50 μm×40 μm was scanned for each composite to map the dispersion of the GNPs using the integrated intensity of the G peak using the spectral range of 1500–1600 cm^−1^. In Figure 12(a)–(c), the color representation was used to distinguish different components: graphene flakes were depicted in blue, and the PMMA matrix was shown in red. Dark-colored regions represented transitions between high intensity (blue) and low intensity (red). These regions indicated the boundaries between graphene and the matrix material, where a shift in intensity from high to low was observed.^46^ In the 0.5GNP-PMMA and 1.0GNP-PMMA samples, the graphene flakes exhibited an even distribution within the PMMA matrix. However, in the 3.0GNP-PMMA sample, a significant amount of graphene flakes overlapped with each other, indicating a high degree of agglomeration. This agglomeration is attributed to the concentration of graphene exceeding the optimal level, leading to the restacking of graphene sheets. The Van der Waals forces between the graphene layers were thought to be responsible for this phenomenon, as reported in the literature.^47^ The mapping process provided insights into the dispersion of GNPs within the composites. It was observed that as the GNP content increased, the size of GNP agglomerates also increased. Figure 12(e) illustrates the relationship between GNP content and the average size of GNP agglomerates. In summary, larger agglomerates of GNPs within the composites are observed with higher GNP content.

The effective load transfer between PMMA and load-carrying graphene plates occurs when the graphene is uniformly dispersed in the matrix.^47^ Graphene nanoparticles and polymer chains share similar size scales, allowing the nanoparticles to penetrate the polymer matrix and interact with the chains. This interaction may enable the GNPs to act as temporary cross-links between the polymer chains, impacting the tensile strength and strain of the GNP-PMMA composite. The incorporation of GNPs into PMMA can result in the formation of a percolating network within the PMMA matrix. This network may enhance the interfacial adhesion between the GNP and the PMMA matrix, promoting strong interactions between the two phases. The GNP's ability to act as temporary crosslinks between the polymer chains in the composite material may have several beneficial effects. These temporary crosslinks create localized regions of increased strength, which can impede the growth of cracks or cavities and enhance the overall tensile strength of the material. By preventing the polymer chains from easily slipping past each other, the crosslinks may effectively immobilize the chains, reducing the material's susceptibility to deformation under the applied stress. As a result of the enhanced interfacial adhesion, uniform stress distribution, and temporary crosslinks provided by the GNPs, the composite material would exhibit increased strain at failure. This means that the material would be capable of undergoing larger deformations or elongations before reaching its breaking point. However, if the distribution of the nanoparticles is not optimal, it can result in regions of weakened strength, reducing the overall performance of the composite. The agglomeration of GNP can also change the morphology and structure of the PMMA, which is an amorphous polymer. For example, it can lead to the formation of interfacial cracks between the nanoparticles and the polymer matrix, which can reduce the adhesion and strength of the composite material. This is likely the case when GNP concentration exceeded 3%. The agglomeration of graphene particles is shown to have an adverse effect on the tensile strength (Figure 2(a)) and strain at fracture (Figure 2(b)) of composites.

**Figure 13.**
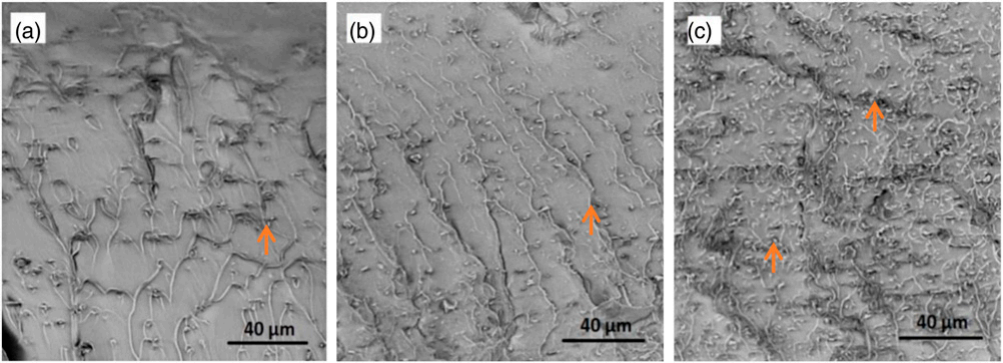
Cryogenic fracture surfaces of (a) 0.5GNP-PMMA, (b) 1.0GNP-PMMA, and (c) 3.0GNP-PMMA composite coatings. Possible cracks at GNP/matrix interfaces are denoted by arrows. PMMA surfaces exhibit meniscus instabilities.

To gain further insights into the formation of defects at the interfaces between graphene and PMMA, cryogenic fracture tests were performed on the composite samples. The resulting fracture surfaces were carefully examined using scanning electron microscopy (SEM), allowing for detailed observations of any existing defects. As shown in Figure 13, debonding at the matrix/graphene interfaces becomes more significant in cases where particle agglomeration occurs at high GNP concentrations. One notable observation is the presence of shear fronts, which were characterized by distinct river-like lines on the fracture surface. These shear fronts are indicative of localized shear stress and are associated with the formation of meniscus instabilities. In the PMMA composite coatings, the graphene particles appear to have arrested the crack fronts, but a higher number of shear fronts are observed in 3.0GNP-PMMA, indicating the formation of defects at high graphene concentrations.

Other changes in polymer structure such as a change in the free volume size and the percentage are possible in the matrices of composites with different GNP concentrations. As the agglomerates can displace or disrupt the polymer chains, changes in the free volume of the polymer may occur creating more space and increasing permeability. The agglomeration of graphene particles has a negative impact on the permeability of composites, as previously shown in Figure 3(a). Compton et al.^48^ proposed that embedding graphene sheets into polymer films could help to densify them by reducing the free volumes within the amorphous polymer matrix. The reduction in free volume can prevent water molecules from permeating through the film, forcing them to take a more tortuous path for diffusion. In the case of the 1.0 GNP-PMMA composite coatings, improved graphene distribution and a lower amount of free volumes, along with a smaller amount of defects at the interfaces, could be cited among the important factors that improved the anti-corrosion performance of PMMA.

However, agglomeration leads to increased defect density and decreased anti-corrosion performance. GNP agglomeration in the composite creates conductive pathways that facilitate ion transport and increase localized corrosion. Galvanic cells form when GNPs contact the metal substrate, accelerating corrosion. Large agglomerated particles facilitate corrosive species movement, reducing graphene's barrier effect. High GNP loads lead to increased defects and diminished anti-corrosion performance.

In summary, the cryogenic fracture surfaces revealed changes in the polymer matrix caused by the addition of GNPs. Changes in the free volume size, improved graphene distribution, and a lower amount of linear defects at the interfaces contributed to the improved anti-corrosion performance of PMMA when 1.0 GNP was added. However, when GNP agglomeration occurs, defect density increases, creating new channels for corrosive media transportation and reducing anti-corrosion performance.

**Figure 14.**
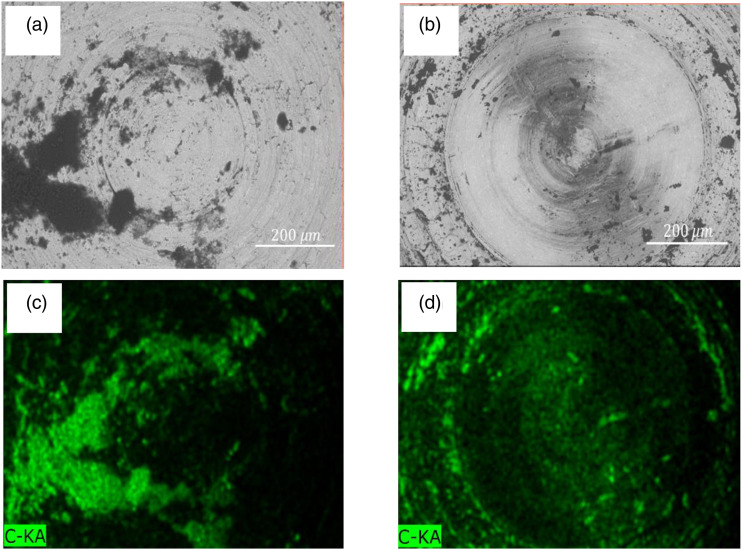
SEM images and associated EDS maps showing the distribution of C on the contact surface of M2 steel pin counterface placed in sliding contact with (a) and (c) neat PMMA, (b) and (d) 3.0GNP-PMMA.

The wear resistance of the composite material was found to increase to its highest level with the incorporation of 1% GNP, but a reduction in wear resistance was observed at higher GNP concentrations. This is consistent with the optimized strength and ductility of the composite at 1% concentration. Interestingly, the coefficient of friction (COF) continued to decrease with increasing GNP concentration, which seems contrary to the overall trend. To explain this, we need to examine the changes at the contact surface of the counterface material. Figure 14(a) and (b) display the secondary electron images (SEI) of the M2 steel pin surface after sliding contact with neat PMMA and 3.0GNP-PMMA composites.

**Figure 15.**
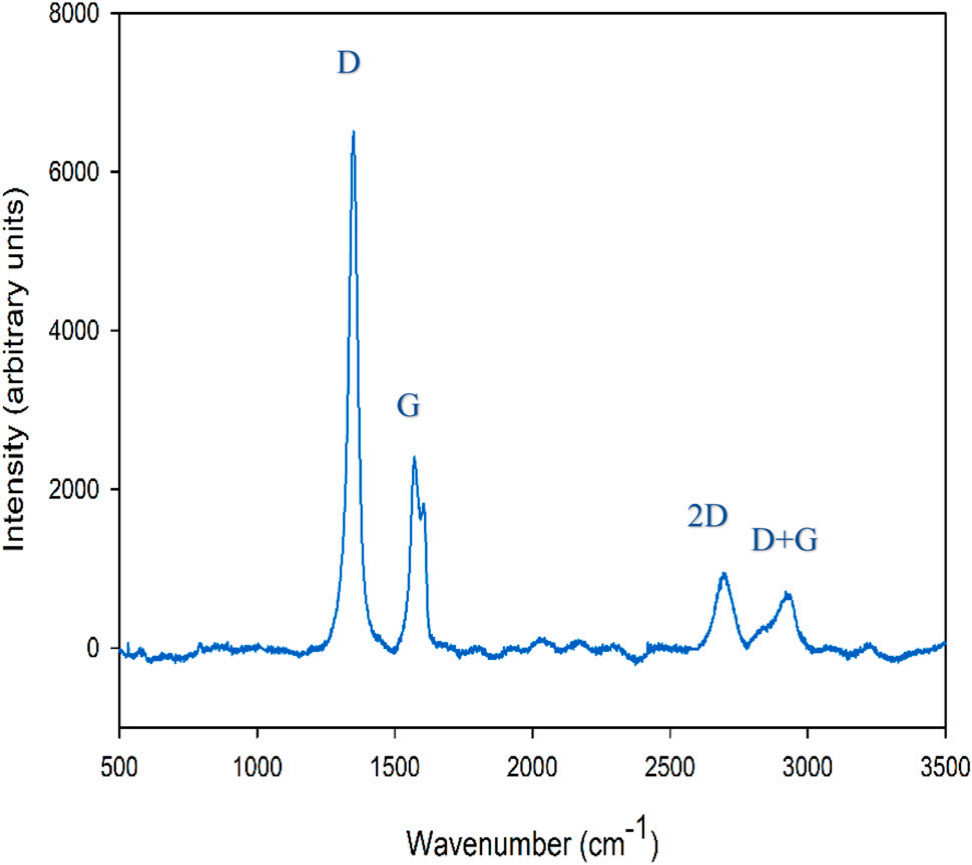
Raman spectra of the transfer layer formed on the contact surface of M2 steel counterface after sliding against 3.0GNP-PMMA composite.

The Raman spectra (Figure 15) obtained from the transfer layers formed on the M2 steel counterface after sliding against 3.0GNP-PMMA, enlightened some key aspects of sliding-induced structural transformations observed. Compared with the Raman spectra of GNPs prior to the sliding test (see Figure 12(d)) it is clear that the intensity of the D peak, which is due to the disorder-induced breathing mode, was considerably increased after the wear test. This suggested an increase in sliding-induced defects. Moreover, a small intensity of the (D + G) band appeared at 2921 cm^−1^ which was not observed in the Raman spectra of GNPs before the wear test. The (D+G) band appearing at 2921 cm^−1^ is a measure of the degree of defects and disorder in the graphene lattice. The appearance of the (D+G) band indicates that the transfer layers had a higher density of defects in graphene leading to increased disorder. The sliding-induced defects may include edge fracture of individual graphene plates and formation of vacancies typically mono-vacancies and Stone-Wales defects would reduce the COF of graphene.

It is important to consider the scale of material transfer events that occur at higher GNP concentrations to understand the frictional behaviour of the composite. At high GNP concentrations, a uniform and well-adhered transfer film is formed. As a result, subsequent sliding occurs between the surface of the GNP-PMMA composite and the transfer layer covering the pin surface, which leads to a reduction in COF. This process is aided by the formation of defects in the transfer layer, which help to reduce the inherent COF of graphene. The defects allow for dissociated water molecules from the atmosphere to weaken the bonds between graphene layers,^44,49^ resulting in further reduction of COF.

## Summary and conclusions

Graphene nanoplatelets (GNP) were incorporated into poly(methyl methacrylate) (PMMA). GNP-PMMA composites were then investigated for their mechanical, electrochemical and tribological performances. The main findings were can be summarized follows:

1. The tensile strength of PMMA increased with the addition of 1 wt % GNP but decreased at higher GNP concentrations. Composites containing ≥3 wt % GNP showed lower fracture strain compared to neat PMMA.
2. Permeability studies revealed that 1 wt % GNP incorporating PMMA resulted in a reduction in the water vapour transmission rate (WVTR), while 3.0GNP-PMMA and 5.0GNP-PMMA showed an increase in WVTR.
3. GNP-PMMA composite coatings had a higher Z-module at 0.01 Hz (|Z|_0.01 Hz_) on Bode plots, indicating an improvement in barrier properties upon the addition of GNP to the matrix for the well-dispersed GNP particles at low concentrations. The Nyquist plot showed that R_coat_ and R_CT_ values were higher in coatings containing 1.0 wt% GNP compared to neat PMMA coatings and coatings with higher GNP concentrations. These results suggest that the incorporation of GNP into the PMMA matrix can enhance the coating's electrochemical performance and increase its resistance to charge transfer.
4. Raman mapping revealed that graphene flakes were evenly distributed in the 0.5GNP-PMMA and 1.0GNP-PMMA samples, while agglomeration was observed in the 3.0GNP-PMMA sample. This agglomeration led to interfacial cracks, affecting mechanical and electrochemical properties and reducing corrosion resistance and water permeability.
5. In the 1.0GNP-PMMA composite, the presence of well-dispersed GNP resulted in high wear resistance by effectively carrying the load and preventing material loss. However, as the GNP concentration increased, the composites exhibited higher wear. Furthermore, defects in the transfer layer containing graphene formed at the tip of the steel pin weakened the bonds between graphene layers, resulting in a reduction in the coefficient of friction (COF) between the surfaces.

In conclusion, future efforts should focus on optimizing the GNP concentration in coating systems to achieve desired mechanical and barrier properties while considering the negative effects of agglomeration. The study emphasizes the importance of understanding interfacial defects and their impact on coating performance. By addressing these aspects, the potential exists to develop enhanced GNP-PMMA composite coatings with improved properties and performance.

## Figures and Tables

**Figure A1.**
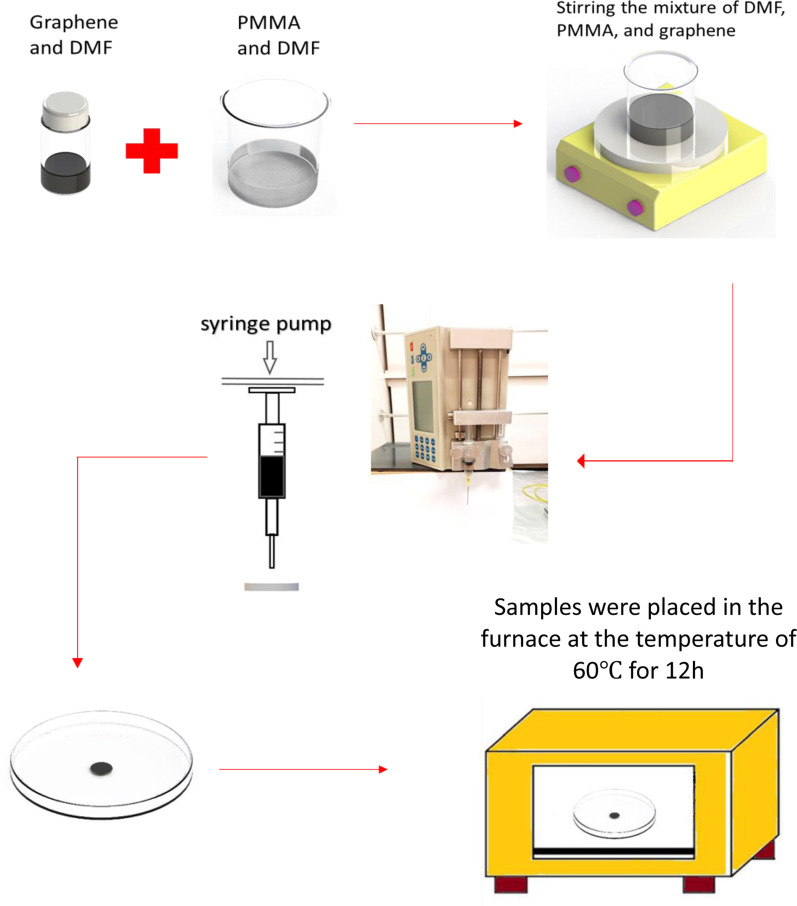
Illustration of solution mixing method used to prepare nanocomposite coatings, and drop-casting method used to deposit the coatings onto the aluminum substrate.
